# Unusual towering elevation of troponin I after ST-elevation myocardial infarction and intensive monitoring with echocardiography post-percutaneous coronary intervention: a case report

**DOI:** 10.1186/1752-1947-4-137

**Published:** 2010-05-18

**Authors:** Fahad Javed, Shahzeb A Khan, Emad F Aziz, Taimur Abbasi, Ramya Suryadevara, Eyal Herzog

**Affiliations:** 1 Division of Cardiology, St. Luke's Roosevelt Hospital Center, University Hospital for College of Physicians and Surgeons of Columbia University, Amsterdam Avenue, 10025, New York, USA

## Abstract

**Introduction:**

The elevation of troponin levels directly corresponds to the extent of myocardial injury. Here we present a case of a robust rise in cardiac biomarkers that correspond to extensive damage to the myocardium but did not spell doom for our patient. It is important to note that, to the best of our knowledge, this is the highest level of troponin I ever reported in the literature after a myocardial injury in an acute setting.

**Case presentation:**

A 53-year-old African American man with an unknown medical history presented to the emergency room of our hospital with chest pain associated with diaphoresis and altered mental status. He required emergency intubation due to acute respiratory failure and circulatory collapse within 10 minutes of his arrival. He was started on heparin and eptifibatide (Integrilin) drips but he was taken immediately for cardiac catheterization, which showed a total occlusion of his proximal left anterior descending, diffuse left circumflex disease and severe left ventricular dysfunction with segmental wall motion abnormality. He remained hypotensive throughout the procedure and an intra-aortic balloon pump was inserted for circulatory support. His urinary toxicology examination result was positive for cocaine metabolites. Serial echocardiograms showed an akinetic apex, a severely hypokinetic septum, and severe systolic dysfunction of his left ventricle. Our patient stayed at the Coronary Care Unit for a total of 15 days before he was finally discharged.

**Conclusion:**

Studies demonstrate that an increase of 1 ng/ml in the cardiac troponin I level is associated with a significant increase in the risk ratio for death. The elevation of troponin I to 515 ng/ml in our patient is an unusual robust presentation which may reflect a composite of myocyte necrosis and reperfusion but without short-term mortality. Nevertheless, prolonged close monitoring is required for better outcome. We also emphasize the need for the troponin assays to be standardized and have universal cutoffs for comparisons across available data.

## Introduction

ST elevation myocardial infarction with elevated cardiac enzymes is a common scenario in emergency rooms. Nowadays, it has become more evident in patients with cocaine abuse. The elevation of troponins directly corresponds to the extent of myocardial injury. We present a case of a robust rise in cardiac biomarkers that correspond to extensive damage to the myocardium but did not spell doom for our patient. It is also important to note how serial echocardiograms in this patient helped us make important decisions regarding his management, all the while keeping in mind his unique cardiac physiology.

Cardiac biomarkers serve as an important and essential component of the initial evaluation of patients with acute coronary syndrome (ACS). Cardiac biomarkers are intracellular macromolecules that are released into the blood circulation due to myocardial injury and are available for detection in the peripheral blood. With the advent of point of care testing and improvement in sensitivity and precision of newer assays, biomarkers not only play a role in diagnosis but also add to prognostic data achieved from history, physical and electrocardiogram (ECG) findings. Like creatine kinase-MB (CK-MB), cardiac troponin I concentrations begin to rise four to six hours after the onset of symptoms and peaks in 18 to 24 hours.

Prospective studies of troponin (cTnI) assays in acute coronary syndromes have demonstrated that cTnI have diagnostic accuracy better than CK-MB [1-3], ECGs [3] and can better predict long-term risk for adverse cardiac events [4-6]. Troponins (I and T) have almost replaced CK-MB as the predominant cardiac biomarker, thus representing cardiac insult since the American College of Cardiology and the European Society of Cardiology redefined the criteria for acute myocardial infarction (MI). However, interpretation of the aggregate data to date is hampered by differences in cutoff values used to define positive tests, the lack of assay standardization (cTnI), the heterogeneity in patient populations to which the tests have been applied, and variations in statistical analysis and presentation of results for cardiac ischemia. In addition, while elevations in troponin I are being interpreted, it is also essential to remember that troponin I can be elevated in conditions other than ACS, such as sepsis, congestive heart failure, renal failure, pulmonary embolism, tachyarrhythmia and myocarditis.

## Case presentation

A 53-year-old African-American man with a medical history of hypertension, smoking and rheumatic fever presented to the emergency room (ER) of our hospital with chest pain. He reported his chest pain to be dull, substernal, non-radiating, lasted over two hours and was associated with diaphoresis. Our patient was in severe respiratory distress with worsening mental status. He was immediately given aspirin, sublingual nitroglycerine and statin, but required emergency intubation due to acute respiratory failure and circulatory collapse on site within 10 minutes of his presentation.

His initial ECG in the ER showed ST elevations of >1.5 mm in leads V1 to V5, ST depressions in leads II and III and VF and Troponin I level of 0.268 ng/ml with CK-MB of 6.1 ng/ml and brain natriuretic peptide (BNP) of 444 pg/ml. He was also started on heparin and eptifibatide (Integrilin) drips, but he was taken immediately for cardiac catheterization, which showed total occlusion of his proximal left anterior descending (LAD), diffuse left circumflex disease and severe left ventricular (LV) dysfunction with segmental wall motion abnormality. A drug eluting stent (DES) was placed in his proximal LAD.

**Figure 1:**
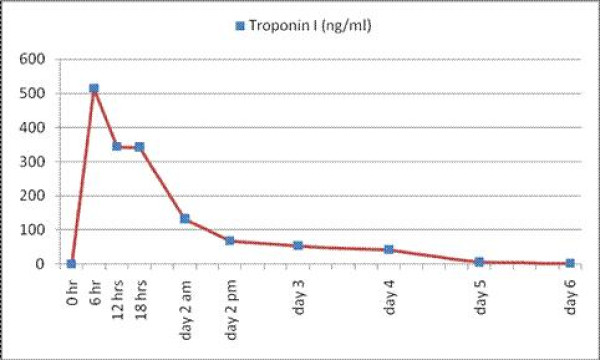
**Trend of our patient's Troponin I level over the first six days of hospitalization**.

Meanwhile, a second set of cardiac enzymes showed troponin I level of 515 ng/ml (Figure 1), CK-MB of 1120 ng/ml, and CK of 11623 ng/ml about six hours after his presentation to the ER. Our patient remained hypotensive throughout the procedure and an intra-aortic balloon pump (IABP) was inserted. He was transferred to Coronary Care Unit for further close monitoring and management.

Subsequently, our patient's urinary toxicology examination result was positive for cocaine metabolites. A transthoracic echocardiogram showed severe LV dysfunction, marked left ventricular hypertrophy, LV ejection fraction of 15%, and akinesia of all his apical segments. His troponins gradually trended down to 42.1 ng/ml on the post-MI day 4, which was a delayed trending course (Figure 1).

Our patient denied chronic cocaine abuse but also mentioned that he was not complying with his home medication of hydrochlorothiazide. At post-MI day six, his IABP medication was discontinued upon careful monitoring of his vitals. Serial echocardiograms confirmed persistent akinetic apex, a severely hypokinetic septum, and severe LV systolic dysfunction. The posterior wall thickness of our patient's heart was 19 mm and an intravenous (IV) septum was found at 19 mm with an apical aneurysm. His visually estimated LV ejection fraction turned into 30% by day 12. Our patient stayed at the Coronary Care Unit for a total of 15 days before he was discharged.

## Discussion

Studies have shown that each increase of 1 ng/ml in the cardiac troponin I level is associated with a significant increase in the risk ratio for death [4]. However, little is known of the mortality risk in patients with troponin levels of 100 ng/ml or above, and how much of aggressive management these patients need after such a myocardial insult. We do know that elevated levels of troponin I provide prognostic information beyond that supplied by the demographic characteristics of patients or even the results of electrocardiogram at their presentation [7].

The elevation of troponin I to 515 ng/ml within six hours of PCI in our patient is an unusual presentation which reflects a composite of myocyte necrosis and reperfusion [8,9]. Many non-randomized [10] and randomized studies [11,12] have confirmed that early coronary intervention attenuates the adverse prognostic impact of troponin elevations [13]. Therefore, the peak of troponin level in our patient can be attributed primarily to an underlying coronary artery disease which was exacerbated by cocaine abuse, rather than secondary to the PCI itself. This hypothesis, however, is still debatable.

The latest American College of Cardiology, American Heart Association and Society for Cardiovascular Angiography and Interventions PCI guideline integrates available data and advocates measurement of biomarkers eight to 12 hours after PCI. Our patient';s elevation of troponin I post-PCI evidently points in favor of these recommendations. However, there is no standard troponin I assay, thus we could not compare threshold values across available studies [14,15]. In addition, we cannot determine which assay is most predictive of outcome. With the availability of highly sensitive assays for the detection of troponins, revised guidelines may be required for diagnostic and prognostic rise and fall in biomarkers in addition to symptoms or ECG changes significant for ischemia and infarction.

## Conclusion

The higher the peak of the troponins after ST segment elevation myocardial infarction (STEMI), the larger the infarct and the higher the risks of complications and death [16,17]. However, the extent of the risk and what should be considered the alarming elevation of troponin is still unclear and needs further exploration. To directly address the actual impact of this variable, future efforts are needed in order to develop standard troponin cutoffs, as well as further data collection across studies, to allow for the combination of study results for pooled analysis or meta-analytic techniques similar to that used by the American College of Cardiology National Cardiovascular Data Registry for cardiac catheterization procedures. If accomplished, this might provide clinicians with a more refined ability to immediately and accurately risk-stratify patients with such high elevation of cardiac biomarkers. Furthermore, it is pointed out that minor post-PCI troponin elevations do not appear to convey a significant short- (or long-) term risk and do not warrant prolongation of hospitalization [18]. However, based on this case report, we assert the need for additional monitoring of patients with elevated cardiac biomarkers through cardiac imaging-like bedside transthoracic echocardiograms for an extended period of time in order to ensure better monitoring, attenuate complications and augment better outcomes.

## Consent

Written informed consent was obtained from our patient for publication of this case report and any accompanying images. A copy of the written consent is available for review by the Editor-in-Chief of this journal.

## Competing interests

The authors declare that they have no competing interests.

## Authors' contributions

FJ and RS were the primary care providers for our patient. EH and EA were the supervising senior cardiologists for all interventions and imaging interpretations. FJ and SAK wrote the manuscript. EH and EA edited the manuscript. All authors read and approved the final manuscript.
